# Myocardial Infarction With Nonobstructive Coronary Arteries: A Significant Adverse Effect of Dihydroergotamine

**DOI:** 10.7759/cureus.82848

**Published:** 2025-04-23

**Authors:** Hong Thoai Nguyen, Juveriya Yasmeen

**Affiliations:** 1 Internal Medicine, Ascension Saint Joseph Hospital, Chicago, USA

**Keywords:** acute coronary syndrome (acs) and stemi, dihydroergotamine, migraine, minoca, myocardial infarction with non-obstructive coronary arteries (minoca)

## Abstract

Dihydroergotamine (DHE), a semi-synthetic ergot alkaloid, has been widely used for decades as an effective treatment for refractory migraines due to its potent vasoconstrictive properties and favorable tolerability profile. Acting primarily through serotonin 5HT1B and 5HT1D receptors, DHE reduces neurogenic inflammation and trigeminal nerve-mediated nociception. However, its broad receptor activity, including alpha-adrenergic, dopaminergic, and serotonergic receptors, also underlies significant cardiovascular risks. In particular, DHE-induced vasoconstriction extends beyond cranial vessels to coronary arteries, potentially leading to serious adverse outcomes such as coronary vasospasm and myocardial infarction. This case report presents a rare instance of DHE-induced myocardial infarction with nonobstructive coronary arteries (MINOCA), emphasizing the importance of recognizing this potentially life-threatening complication. Despite long-term use without prior adverse effects, our patient developed MINOCA after receiving DHE to treat migraine attacks, highlighting the unpredictable nature of DHE's vasoconstrictive effects and underscoring the need for heightened clinical vigilance, especially in patients with underlying cardiovascular risk factors. Given the diagnostic and prognostic ambiguity surrounding MINOCA, this case contributes to the growing body of literature advocating for individualized risk assessment and cautious DHE administration.

## Introduction

Dihydroergotamine (DHE) has long been employed as a therapeutic agent for refractory migraines due to its potent vasoconstrictive properties [1]. Despite its efficacy, the cardiovascular risks associated with DHE remain underappreciated. Specifically, DHE induces vasoconstriction in both cranial and coronary arteries, which can precipitate severe cardiovascular events. Although DHE-induced coronary vasospasm is rare, it carries the risk of causing potentially fatal acute myocardial infarction. Myocardial infarction with nonobstructive coronary arteries (MINOCA) is a clinical syndrome characterized by evidence of myocardial infarction without significant coronary artery stenosis on angiography [2]. MINOCA represents a heterogeneous clinical syndrome characterized by a spectrum of underlying etiologies. These include epicardial coronary pathologies such as plaque rupture, coronary artery spasm, and spontaneous coronary artery dissection, as well as microvascular abnormalities, including coronary microvascular spasm, coronary thromboembolism, Takotsubo (stress-induced) cardiomyopathy, and myocarditis [2].

Chest pain is the most prevalent presenting symptom in patients with suspected acute coronary syndrome, with a reported prevalence of approximately 92% [3,4]. Nevertheless, atypical manifestations such as neck pain, back pain, throat discomfort, otalgia, and hiccups are not uncommon [5]. Notably, craniofacial pain may be the sole presenting symptom in up to 6% of individuals with acute myocardial infarction [6]. In this case report, we describe a patient who, after being treated with DHE for refractory migraines, developed MINOCA, highlighting the critical need for awareness of the cardiovascular risks associated with DHE, particularly in patients with underlying cardiovascular risk factors.

## Case presentation

A 58-year-old female patient with a medical history of migraines, hypertension, a ruptured cerebral aneurysm, hypercholesterolemia, type 2 diabetes mellitus, asthma, and pituitary microadenoma with hyperprolactinemia was admitted to the hospital due to worsening migraine headaches. She had previously been hospitalized for severe migraine headaches and had been using DHE since 2014 without experiencing any adverse effects. Her current medication regimen included carvedilol, candesartan, rosuvastatin, ezetimibe, DHE nasal spray, erenumab-aooe, and ubrogepant. Furthermore, a nuclear stress test conducted six months prior to this admission had yielded negative results for inducible myocardial ischemia or infarction.

**Figure 1:**
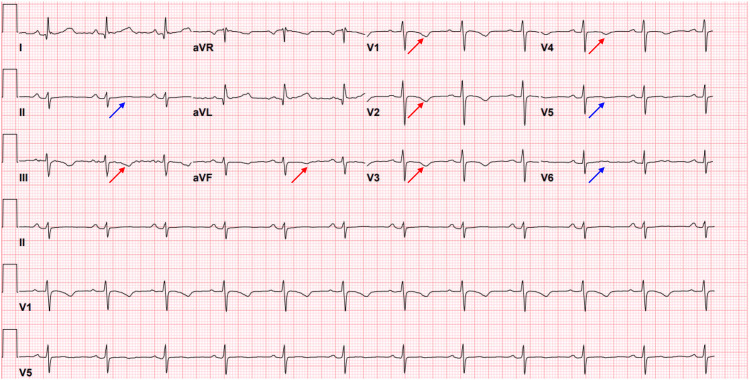
The ECG showed symmetrical T wave inversion on III, aVF, and V1-V4 (red arrows) and a flat T wave on II, V5, and V6 (blue arrows). No ST elevation was noted, and the QTc was 488 milliseconds.

Upon admission, the patient received two doses of intravenous DHE 0.5 mg. Six hours after the second dose, the patient experienced hypotension with a blood pressure of 60/40 mmHg and a pulse of 77 bpm. She exhibited drowsiness, ongoing headaches, dizziness, and lethargy. Although she initially denied chest pain, shortness of breath, or palpitations, five hours later, she reported pressure-like chest pain on the left side radiating to her back. The ECG showed normal sinus rhythm with a heart rate of 76 bpm, symmetrical T wave inversion on III, aVF, V1-V4, and a flat T wave on II, V5, and V6. No ST elevation was noted, and the QTc was 488 milliseconds (Figure 1). The high-sensitivity troponin initially peaked at 4838 pg/ml and subsequently decreased to 2512 pg/ml. The creatinine level was 1.24 mg/dl, and the D-dimer was 372 ng/ml (Table 1). A CT angiogram of the chest did not reveal aortic dissection but did show severe coronary artery calcifications in the LAD. The echocardiogram indicated a normal left ventricular ejection fraction (LVEF) of 60% with no segmental hypokinesia, dyskinesia, or akinesia.

**Table 1:** The patient's high-sensitive troponin, creatinine, and D-dimer.

Component	Reference range	At the time of chest pain	Six hours later
High-sensitive troponin (pg/ml)	0-12	4838	2512
Creatinine (mg/dl)	0.6-1.2	1.24	
D-dimer (ng/ml)	0-500	372	

**Figure 2:**
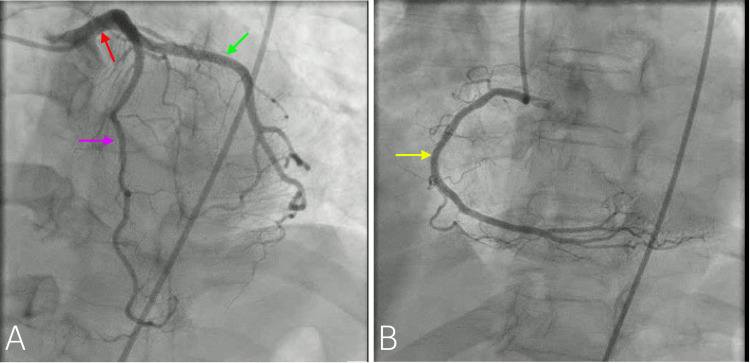
The coronary angiogram revealed a normal left main (red arrow), 10% smooth calcified LAD stenosis (purple arrow), normal CX system (green arrow), 10% smooth RCA stenosis (yellow arrow). A: Angiography of left anterior descending and left circumflex arteries B: Angiography of right coronary artery LAD: left anterior descending; CX: circumflex; RCA: right coronary artery

The patient was diagnosed with non-ST elevation myocardial infarction (NSTEMI) and received aspirin and heparin infusion. An urgent coronary angiogram and possible percutaneous coronary intervention (PCI) were planned. The coronary angiogram revealed a normal left main, 10% smooth calcified left anterior descending (LAD) stenosis, normal circumflex (CX) system, 10% smooth right coronary artery (RCA) stenosis, and LVEF of 60% (Figure 2). No culprit vessel was identified, leading to the inference that DHE vasospasm was the likely cause. The cardiac MRI or vasospasm provocation studies were not performed. Following the angiogram, the patient was prescribed aspirin, metoprolol, rosuvastatin, ezetimibe, and candesartan. DHE was completely discontinued, and her chest pain significantly improved and was resolved the following day.

## Discussion

DHE, a semi-synthetic derivative of ergotamine, has been utilized for over 70 years to suppress vascular headaches, particularly for the abortive treatment of acute migraine headaches. Its superior tolerability profile has led to its widespread preference over ergotamine despite both compounds having comparable antimigraine effects [1].

DHE primarily acts as an agonist at serotonin 5HT1B and 5HT1D receptors. It also exhibits agonistic activity at 5HT1A, 5HT1F, 5HT2A, 5HT2C, 5HT3, 5HT4, ɑ1, ɑ2, ꞵ, muscarinic, D1, and D2 receptors. Vasoconstriction is facilitated through the 5HT1B receptor, while its antimigraine effects are attributed to the 5-HT1D receptor, which reduces the release of neurogenic inflammatory agents such as calcitonin gene-related peptide and disrupts trigeminal nerve-mediated nociception [7-9].

DHE acts as an alpha-adrenoreceptor blocker and directly stimulates the smooth muscle in peripheral blood vessels. It has a more pronounced effect on capacitance vessels (veins) compared to resistance vessels (arterioles). DHE is more potent than ergotamine in its adrenergic blocking actions and less potent in producing arterial vasoconstriction while still maintaining a significant venoconstrictor effect [10,11].

The vasoconstrictive effects of DHE are cranioselective but also lead to greater vasoconstriction in the distal cardiac arteries than in the proximal ones. Furthermore, DHE diffuses slowly from its receptors, resulting in prolonged vasoconstrictive effects [12]. Therefore, the use of DHE is not recommended for individuals with cardiac disease (uncontrolled hypertension, ischemic heart disease, angina, history of MI, and coronary vasospasm), peripheral vascular disease, as well as severe hepatic and renal dysfunction due to concerns about potential cerebral, cardiac, or peripheral ischemia caused by vasoconstriction [13].

MINOCA is defined by the presence of myocardial infarction with normal or near-normal coronary arteries on angiography (stenosis < 50%). MINOCA encompasses a diverse range of conditions with varying underlying pathophysiological mechanisms, thus raising questions about the appropriateness of applying conventional secondary prevention and treatment strategies developed for myocardial infarction with obstructive coronary artery disease (MI-CAD) to MINOCA patients. The prognosis and prognostic indicators for MINOCA patients are not yet fully understood. While MINOCA patients generally have a slightly more favorable prognosis compared to MI-CAD patients, MINOCA is not uniformly benign and can, in some cases, lead to fatal outcomes [2].

The patient presented with multiple cardiovascular risk factors, including hypertension, hypercholesterolemia, and type 2 diabetes mellitus. A previous negative nuclear stress test ruled out inducible myocardial ischemia or infarction. The patient had been using DHE since 2014 without any adverse effects. However, upon administration during this admission, the patient experienced hypotension and chest pain, with significantly elevated high-sensitivity troponin levels and ECG findings suggestive of NSTEMI. Coronary angiography revealed normal coronary arteries with minimal stenosis and a LVEF of 60%. No culprit’s vessel was identified. It was concluded that DHE caused vasoconstriction in the coronary arteries, resulting in MINOCA. The patient discontinued DHE while maintaining aspirin, metoprolol, rosuvastatin, ezetimibe, and candesartan therapy.

The occurrence of DHE-induced coronary vasospasm leading to MINOCA is a rare phenomenon in clinical practice. Although there are reservations about administering DHE to patients with underlying cardiac conditions such as uncontrolled hypertension, ischemic heart disease, angina, history of myocardial infarction, and coronary vasospasm, our patient has been using it since 2014 without experiencing any adverse effects. This suggests that the vasoconstrictive properties of DHE are unpredictable and can vary from individual to individual. The prognosis of MINOCA varies based on its causes and remains ambiguous, although it is marginally more favorable for MINOCA patients compared to those with myocardial infarction and coronary artery disease. In certain instances, DHE-induced MINOCA can have fatal consequences [14-16].

Multiple reports have documented fatal myocardial infarction attributed to vasospastic events associated with DHE administration. In particular, two cases involved patients without significant coronary artery stenosis who succumbed to acute myocardial infarction while receiving subcutaneous DHE (0.5 mg twice daily) in combination with 2,500 IU of heparin [17]. Furthermore, a prospective study identified two deaths occurring in the high-dose DHE cohort [18]. In contrast, prompt recognition and appropriate management in our patient led to a favorable outcome, averting mortality and preventing further complications.

## Conclusions

This case highlights a rare but significant cardiovascular complication associated with DHE use MINOCA in a patient with multiple cardiovascular risk factors but no prior adverse response to long-term DHE therapy. Despite its clinical efficacy and historical safety profile, DHE's potent vasoconstrictive effects can unpredictably extend beyond cranial vessels to coronary circulation, leading to potentially life-threatening outcomes. This underscores the importance of heightened clinical vigilance and individualized risk assessment when prescribing DHE, especially in patients with pre-existing cardiovascular comorbidities. Clinicians should maintain a high index of suspicion for coronary vasospasm and consider alternative migraine therapies in high-risk individuals. This case also highlights the need for future research, prescribing guidelines, and pharmacovigilance practices. Early recognition, discontinuation of the offending agent, and appropriate medical management can significantly improve patient outcomes in cases of DHE-induced MINOCA.
